# A Sustained Dietary Change Increases Epigenetic Variation in Isogenic Mice

**DOI:** 10.1371/journal.pgen.1001380

**Published:** 2011-04-21

**Authors:** Cheryl C. Y. Li, Jennifer E. Cropley, Mark J. Cowley, Thomas Preiss, David I. K. Martin, Catherine M. Suter

**Affiliations:** 1 Victor Chang Cardiac Research Institute, University of New South Wales, Sydney, Australia; 2 Faculty of Medicine, University of New South Wales, Sydney, Australia; 3 Peter Wills Bioinformatics Centre, Garvan Institute of Medical Research, Sydney, Australia; 4 Cancer Research Program, Garvan Institute of Medical Research, Sydney, Australia; 5 Faculty of Science, University of New South Wales, Sydney, Australia; 6 Children's Hospital Oakland Research Institute, Oakland, California, United States of America; The University of North Carolina at Chapel Hill, United States of America

## Abstract

Epigenetic changes can be induced by adverse environmental exposures, such as nutritional imbalance, but little is known about the nature or extent of these changes. Here we have explored the epigenomic effects of a sustained nutritional change, excess dietary methyl donors, by assessing genomic CpG methylation patterns in isogenic mice exposed for one or six generations. We find stochastic variation in methylation levels at many loci; exposure to methyl donors increases the magnitude of this variation and the number of variable loci. Several gene ontology categories are significantly overrepresented in genes proximal to these methylation-variable loci, suggesting that certain pathways are susceptible to environmental influence on their epigenetic states. Long-term exposure to the diet (six generations) results in a larger number of loci exhibiting epigenetic variability, suggesting that some of the induced changes are heritable. This finding presents the possibility that epigenetic variation within populations can be induced by environmental change, providing a vehicle for disease predisposition and possibly a substrate for natural selection.

## Author Summary

Epigenetic changes to gene expression that do not involve changes to DNA sequence can be influenced by the environment and provide one candidate mechanism by which early nutrition can influence adult disease risk. Here, we examined epigenetic changes across the genome in response to short- and long-term exposure to a dietary supplement in genetically identical mice. We find that the supplement induces small but widespread epigenetic changes in exposed mice. These changes increase the epigenetic variability among exposed mice, and this effect is magnified in mice exposed long-term. The epigenetic changes are overrepresented in gene functions involved in cell and organ development and in gene expression. Our data is consistent with the external environment having pervasive effects on the epigenome and suggests that some genetic pathways may be more susceptible to environmental influence than others.

## Introduction

Epigenetic modifications lie at the interface between genes and the environment, and thus have the potential to create functional diversity in response to environmental cues. There is mounting evidence that the establishment of epigenetic states during mammalian development can be influenced by the gestational and neonatal milieu, resulting in lifelong phenotypic changes. Epigenetic changes have been observed after early exposure to a variety of insults including environmental toxins [1], variations in maternal care [2], *in vitro* culture [3] and nutritional stressors [4]–[12]. In some cases the epigenetic effects are heritable, giving rise to environmentally-induced phenotypes in subsequent, unexposed generations [1], [5].

The epigenetic response to altered nutrition is of great interest because it may explain how nutritional stress during gestation can have health effects beyond the neonatal period. Suboptimal nutrition or exposure to environmental toxins or stress during gestation increases the susceptibility of offspring to a number of adult-onset diseases, a phenomenon known as fetal programming [13]. It has been widely speculated that epigenetic changes underlie the phenotypic response to early nutritional stress [14]–[17], but the genes responsible for the phenotypic changes are not known, and few studies have examined the magnitude and extent of epigenetic changes in response to altered nutrition.

Perhaps the best-studied model of epigenetic response to nutrition is the effect of methyl donor supplementation on the murine *A^vy^* allele. Supplementation of pregnant dams with methyl donors influences the epigenetic state of the *A^vy^* allele in offspring, resulting in suppression of the obese yellow phenotype characteristic of *A^vy^* mice [4]–[5], [9]. We have previously shown that this environmentally-induced epigenetic change can be passed from one generation to the next [5]. However, there is no reason to suppose that the *A^vy^* allele is the only locus whose epigenetic state is susceptible to dietary influence. Epigenetic changes have been observed at various individual loci after exposure to general nutritional deprivation or excess [7], [18]–[21] and more recent genome-wide screens in cases of intrauterine growth restriction have suggested that changes may occur at loci throughout the genome [22]–[23].

We have investigated the extent of epigenetic changes induced by methyl donors, by assessing cytosine methylation at CpG island promoters across the genome in mice exposed to methyl donors for one or six generations. We find that methyl donors induce stochastic changes in methylation at thousands of loci throughout the genome, leading to an increase in epigenetic variability among individuals that is more pronounced in mice exposed for multiple generations. While affected genes differed among individual mice, similar functional groups were affected: genes involved in gene expression and transcription, organogenesis, and cellular development were highly overrepresented, suggesting that these genetic programs may be more susceptible to environmental influence.

## Results

In order to assess the extent of epigenetic changes in response to dietary methyl donors, we examined changes in DNA methylation across the genomes of isogenic C57Bl/6J mice. Dietary supplementation with methyl donors commenced in founder pairs two weeks prior to mating, and was continued throughout pregnancy and lactation. We collected hepatocytes for analysis from mice in the first generation of exposure, and after supplementation for six generations. These mice were compared with C57Bl/6J mice that had never been exposed to methyl donors.

### Methyl donors do not alter global 5-methylcytosine levels

**Figure 1:**
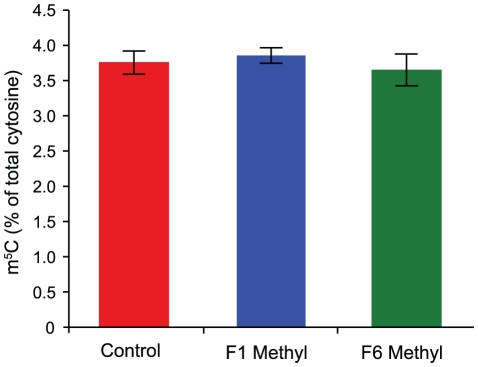
Methylation levels are unchanged after methyl donor supplementation. Whole-genome 5-methylcytosine (m^5^C) content in liver DNA from control, F1 supplemented (F1 Methyl) and F6 supplemented (F6 Methyl) mice as assessed by HPLC (n = 5 per group). Error bars indicate standard deviation.

Methyl donors participate in an arm of one-carbon metabolism that creates methyl groups for donation to various molecules, including DNA, via the conversion of S-adenosylmethionine to S-adenosylhomocysteine. The observed effect of methyl donors on the *A^vy^* allele – epigenetic silencing of the IAP element that drives ectopic expression of the agouti gene [4]–[5], [9] – has been supposed to result from increased cytosine methylation due to an increase in the availability of methyl groups [9]. To determine if methyl donor supplementation leads to a global increase in the level of cytosine methylation, we assessed 5-methylcytosine (m^5^C) levels in genomic DNA from the livers of supplemented and unsupplemented mice by high-performance liquid chromatography (HPLC). We find that the m^5^C content of DNA from supplemented mice is not increased, even after six generations of supplementation (Figure 1).

### Epigenetic variability is increased by methyl-donor supplementation

The absence of gross changes in genomic m^5^C levels does not preclude changes at some loci in supplemented mice. Methyl donors have been reported to induce epigenetic changes in at least two discrete loci (*A^vy^* and *Axin^Fu^*) [5], [12] but it is not known if other genomic loci are also affected. To determine whether methyl donors exert epigenetic changes at other loci, and to resolve the extent of any changes, we compared genomic methylation patterns of supplemented and unsupplemented mice using a recently described method that combines enrichment of the unmethylated fraction of DNA with promoter microarray analysis [24]. Enrichment of the unmethylated fraction gives a better signal-to-noise ratio than other methods based on enrichment of methylated DNA, because removal of most repetitive sequences reduces the size of the DNA pool; moreover, since unmethylated CpG dinucleotides are less abundant in the genome than methylated CpG dinucleotides, this method is considerably more sensitive to DNA methylation changes at CpG islands [25].

We constructed libraries enriched for the unmethylated fraction of genomic DNA from liver using sequential HpaII and McrBC digestion and ligation-mediated PCR [24], and hybridised them to Agilent Mouse CpG Island 105K arrays representing approximately 16,000 CpG islands. We chose to examine CpG islands for two reasons: first, methylation changes at CpG islands are more likely to reflect regulatory changes than methylation changes at low-CpG density loci [26]; second, the enzymatic enrichment method we used preferentially targets CpG islands. We compared libraries from five F1 and five F6 supplemented mice to those from five unsupplemented controls; pooled libraries from 10 unsupplemented controls acted as the reference sample for each array. We analysed normalised array data using Partek Genomics Suite software.

**Figure 2:**
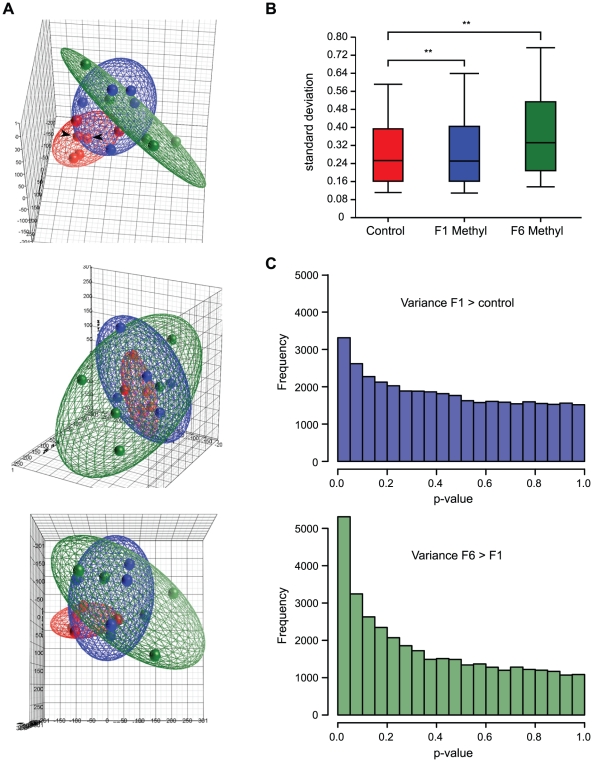
Methyl donor supplementation increases epigenetic variation in exposed mice. (A) Pseudo three-dimensional plot showing principal components analysis (PCA) of microarray data from control (red) and F1 (blue) and F6 (green) supplemented mice. The same plot is shown from three different perspectives. The ellipsoids around the PCA scores of each group were determined by standard deviations, so that their size is indicative of the overall variance within the group. (B) Box-and-whisker plots showing distribution of standard deviation values of intra-group log Cy3/Cy5 ratios across all microarray probes. Whisker lines indicate 90th and 10th percentile values. ** = *p*<0.0001. (C) Frequency histogram showing the number of probes with the given probabilities of higher variance (upper panel) in F1 supplemented than control animals, and (lower panel) in F6 than F1 supplemented animals. The accumulation of probes with small p-values indicates that more probe signals are significantly more variable in F1 than control, and F6 than F1.

To view the overall distribution of array data from each group of mice, we performed a principal component analysis (PCA). PCA is a variable reduction procedure by which data with many variables is reduced to a few artificial variables, called principal components, which together account for most of the variance in the actual variables. The first three components of our data accounted for 38.7% of the variability and are visualized as a pseudo three-dimensional score plot in Figure 2A. In this visualization, array datasets from control mice cluster more closely than datasets from supplemented mice, suggesting that there is less variability between datasets from control animals than between those from supplemented animals. But control datasets do not overlap each other entirely, showing that there is some variability between controls. This variability cannot be attributed to technical variation between arrays, as principal component scores from array replicates were highly similar, so it is most likely due to methylation differences between control animals. This suggests that isogenic mice exposed to the same environment exhibit intrinsic epigenetic variation.

To confirm that the inter-individual epigenetic variation we observed was indeed biological in origin and not due to some intrinsic variability in probe signal, we measured the intrinsic variability of each probe by calculating the standard deviation of the signals from the reference pool across all 15 arrays. We compared this value with the probe's array signal standard deviation in each group. We found no correlation between reference pool standard deviation and array signal standard deviation (Figure S1). We also find no correlation between array signal standard deviation and probe GC content, which is the primary source of intrinsic variation in probe hybridization behavior [27] (Figure S1). This data indicates that the inter-sample variation we observe is due not to technical variation, but rather to methylation differences between animals.

Array datasets from supplemented mice show a broader range of principal component scores than those from controls (Figure 2A), indicating that array data from supplemented mice are more variable. Datasets from supplemented mice are also spatially distinct from control datasets in the PCA. Together, this suggests that supplemented mice have methylation patterns that are both more variable than, and different from, unsupplemented mice. Principal component scores from F6 supplemented animals show even greater dispersal than those from F1 animals, suggesting that the increased variability in methylation patterns seen in methyl donor supplemented animals is amplified with multigenerational exposure. Datasets from long-term supplemented mice are also more distant from controls than those from short-term supplemented mice. This suggests that in addition to increasing methylation variability, long-term supplementation may cause mice to become progressively more epigenetically distinct from mice that have never been supplemented.

As a second measure of overall variability in the array data, we calculated the range of probe signal standard deviations within each treatment group (Figure 2B). The average standard deviation was significantly higher for both F1 and F6 supplemented mice than for controls (*p*<0.001, unequal variance t-test), consistent with greater variability in methylation patterns between individual supplemented mice than between individual controls.

**Figure 3:**
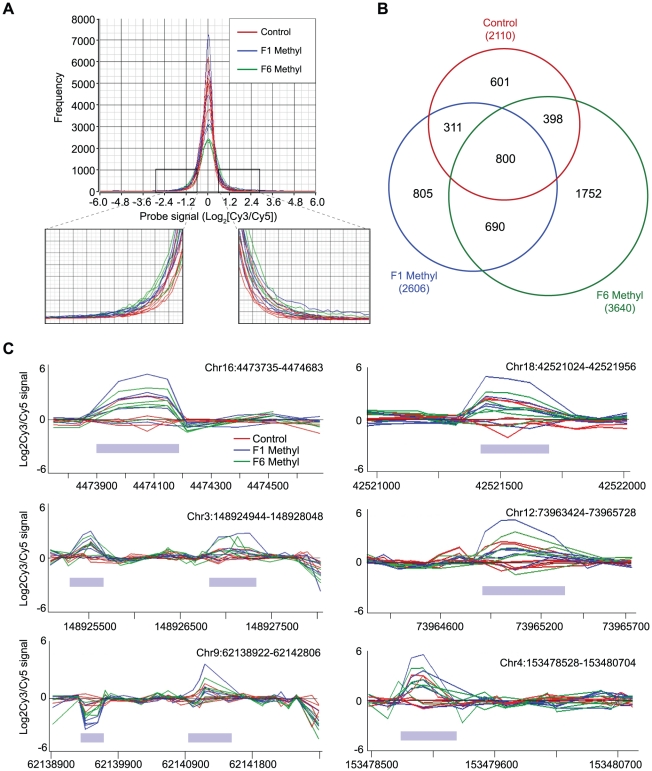
Methylation-variable regions in unsupplemented and supplemented mice. (A) Histogram showing frequency distribution of normalised array probe signals from control and F1 and F6 supplemented mice. The areas of the histogram showing the lowest and highest signals (representing the greatest losses or gains in methylation relative to the control pool) are magnified. (B) Venn diagram showing overlap of loci identified as methylation-variable between unsupplemented and F1 and F6 supplemented mice. The total number of methylation-variable loci in each group is shown in parentheses. (C) Microarray signals from six representative methylation-variable loci. Note that an increase in signal indicates relative *hypo*methylation. Grey bars indicate methylation-variable regions.

Third, we analysed each probe to determine whether it was more variable in one treatment group than another (Bartlett's test): this revealed significantly more variability in short term supplemented mice than control mice, and in long term than short term supplemented mice (Figure 2C). Finally, consistent with the idea that methyl donor supplementation increases epigenetic variability, histogram plots of array signals show an increased frequency of very low and very high signals in exposed mice (Figure 3A). Taken together, these results indicate that supplemented mice harbor many loci that carry more or less methylation relative to control mice.

### Methylation changes at individual loci are stochastic among individuals

The measures that we performed indicated variability in methylation at individual CpG island loci in the genomes of both unsupplemented and supplemented mice. To identify candidate changes at individual loci induced by methyl donor supplementation, the conventional approach would be an analysis of variance (ANOVA). But candidate identification by ANOVA relies on within-group variance being lower than between-group variance, and our measures of overall variability indicated high within-group variance (particularly within the supplemented groups). Thus an ANOVA of our datasets yielded very few candidate loci, which when subjected to validation by extensive bisulphite sequencing showed no change in methylation (data not shown). We therefore took a different approach and first attempted to identify where methylation variability occurs, regardless of the treatment group: to do this, we interrogated the array probes that showed the most variable signals between mice of the same group, rather than between groups.

We identified probes with standard deviation values above the 95^th^ percentile of the control group and mapped them to their respective CpG islands; we arbitrarily defined these loci as “methylation-variable”. We find 2110 methylation-variable loci in the control group, 2606 in F1 and 3640 in F6 (Figure 3B; for a list of all methylation-variable loci, see Table S1). There were 1490 methylation-variable loci in common between the short-term and long-term supplemented groups; 800 of these were also methylation-variable in the controls. A considerable proportion of methylation-variable loci were unique to each treatment group: long-term supplemented animals display the most (1752 or 48% of all this group's methylation-variable loci) and control animals the least (601 or 28%). Thus, not all the loci that are methylation-variable in control animals were affected by methyl donors in our sample supplemented population; this may be a reflection of the small sample size.

Representative methylation-variable loci are illustrated in Figure 3C. The variable regions are tightly defined and are flanked by sequence that is methylation-invariant among animals. Consistent with our finding that methyl donors do not alter global levels of m^5^C, we find that methylation-variable loci in supplemented animals are as likely to lose methylation as to gain it (Figure 3A and 3C). This challenges the assumption that methyl donors exert epigenetic effects via an increase in cytosine methylation [7], [9], and is consistent with our previous finding that methyl donors increase the probability of silencing at *A^vy^* without increasing the level of cytosine methylation [28]. At any given methylation-variable region, differences invariably occur in the same direction, although the amplitude differs among mice. Four loci interrogated by bisulphite allelic sequencing are shown in Figure S3. We found that just over half of validated loci (5/9) showed small methylation changes in the direction indicated by the array; the verification rate (FDR ∼0.55), and the small magnitude of changes we observe, are comparable to that of previous studies using this array strategy [29]–[30].

Taken together these results show that methylation variability occurs at many loci across the genomes of isogenic mice, and that the number of loci that exhibit variability increases with exposure to dietary methyl donors. Methylation changes in response to methyl donors are therefore stochastic and act to increase the epigenetic variability extant in an isogenic population.

### Genes associated with methylation-variable loci are overrepresented in developmental ontologies

**Figure 4:**
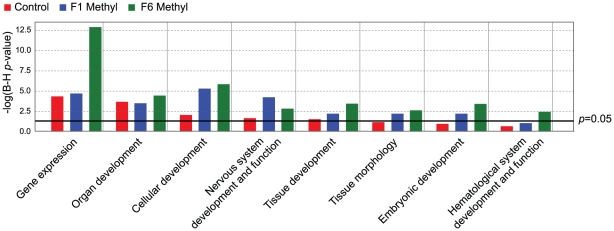
Gene ontology analysis of methylation-variable genes in unsupplemented and supplemented mice. Graph from IPA showing gene ontology categories significantly overrepresented in the genes defined as methylation-variable in control and F1 and F6 supplemented mice. The black line indicates a significance threshold of *p* = 0.05 with Benjamini-Hochberg correction for multiple testing.

We find significantly more methylation-variable loci that are common to the three groups than expected by chance (800 vs 150; *p*<0.0001, χ^2^ test, 6 degrees of freedom); this suggests that methylation variability does not occur randomly, but rather that some genes are more epigenetically “plastic” than others. We performed a gene ontology (GO) analysis of the methylation-variable loci using two independent methods (Ingenuity Pathways Analysis (IPA) and GOstat [31]), to determine whether genes associated with these loci had functions in common. Both methods showed that genes involved in transcription, development and organogenesis are significantly overrepresented in methylation-variable loci, and that this is independent of dietary intervention (Figure 4 and Table S2). This applied to the loci that were common among groups as well as those unique to a group; thus, although genes may be idiosyncratically methylation-variable from one individual to the next, the variations appear to occur in common pathways.

### Methylation variability is independent of local sequence characteristics

We considered the possibility that the methylation variability we observed was conditioned by the underlying genetic sequence, and so compared the sequence composition of the promoter regions (−1000 bp to +500 bp relative to the TSS) associated with the 100 most variable probes in the control group to that of the promoters associated with the 1000 least variable probes. We found no difference in GC content between methylation-variable and methylation-invariant promoters (Figure S2). We ran a *de novo* motif prediction pipeline (GimmeMotifs) to uncover any DNA motifs common to variable promoters, then compared the frequency of these motifs between the methylation-variable and methylation-invariant promoters. We identified nine motifs in the promoters of variable genes, but none of these were enriched relative to the methylation-invariant set (data not shown). Finally, given the known role of repetitive elements in affecting the epigenetic state of nearby genes, we examined the frequency and relative location of genomic repeat elements (LINE, SINE, LTR retrotransposons, simple repeats, low complexity repeats, microsatellites and DNA transposons) in the same promoter regions as above. We found no evidence for a difference in either repeat frequency or distribution between methylation-variable and methylation-invariant promoters (Figure S2). Taken together, these results indicate that local sequence context is unlikely to account for the methylation-variable regions that we have observed.

## Discussion

We have conducted a genomewide DNA methylation analysis to investigate the epigenomic consequences of a sustained nutritional change, methyl donor supplementation. The epigenetic effect of dietary methyl donors has been well documented at the retrotransposon-derived murine *A^vy^* allele, but the extent to which the genome as a whole is affected by any sustained dietary intervention is largely unexplored. We found that methyl donor supplementation has widespread effects which increase epigenetic variation and are exacerbated by long-term exposure.

The increase in epigenetic variation induced by methyl donors occurred on a background of inter-individual epigenetic variation already extant in C57BL/6J mice. DNA from different control mice did not give identical array signals; these differences cannot be attributed to technical variation or genetic differences, and indicate epigenetic variation between isogenic mice reared in the same environment. The methylation-variable regions we defined usually do not span entire CpG islands, but are restricted to a subset of probes within each affected island, with surrounding probes showing no variability. Since the CpG islands on the array were chosen using computational (rather than functional) criteria, the methylation-variable regions we have identified may represent functional components within CpG islands. Our finding of well-defined methylation-variable loci in a control population of isogenic individuals is consistent with previous observations of variably methylated regions (VMRs) in the genomes of inbred mice by Feinberg and Irizarry [32]. Although the two studies used different methods of analysis, they identified methylation-variable regions that show striking overlap in gene ontology. It would be interesting to examine whether the widespread epigenetic differences that have been observed between human monozygotic twins [33]–[34] occur in genes from the same ontologies.

**Figure 5:**
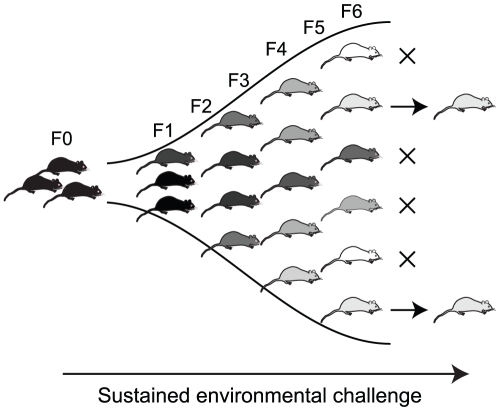
Epigenetic diversity induced by sustained exposure to environmental change as a substrate for natural selection. In this model, an environmental change (such as methyl donor supplementation) increases epigenetic variability between individuals, with cumulative effects over generations. Over time, individuals become epigenetically distinct from the original population, and also from each other. This epigenetic variation leads to increased variability in phenotype, on which selection may act.

While several independent studies (including this one) now suggest that epigenetic variation persists in the absence of any genetic or environmental change, this study provides the first indication that additional epigenetic variation can be induced by environmental exposure. Methyl donor supplementation resulted in an increase in the number of methylation-variable loci: the epigenetic changes induced by dietary methyl donors were small in magnitude but widespread throughout the genome. Importantly, changes were stochastic, occurring at different loci in different individuals. Long-term exposure to excess methyl donors further increased the epigenetic variability within the population. That the effect becomes more pronounced with multigenerational exposure suggests that at least some of the induced changes are heritable. If so, phenotypic diversity created by an environmentally-induced increase in epigenetic variability might be acted upon by natural selection independently of genotype (Figure 5). This could enable rapid (within a few generations) adaptation to new environments [35]–[37], and because no genetic change is required, the acquired phenotypes would potentially be reversible if environmental conditions reverted. A sustained environmental change over a longer period might eventually result in a permanent epigenetic change which can in turn facilitate genetic mutation through the increased mutability of 5-methylcytosine [32], [38]–[39].

The idea that nutritional perturbations result in epigenetic changes throughout the genome, as opposed to at a few key regulatory genes, is consistent with the findings of several recent studies investigating the epigenetic contribution to fetal programming. Most candidate-approach studies report small, subtle methylation changes (typically <10%) [7], [19], [21]–[23]; reports of larger changes are less common [40]–[41]. An immediate question that arises is whether such small methylation changes are likely to exert any significant effect on phenotype. The VMRs identified by Feinberg and Irizarry were associated with gene expression variability [32], so small methylation changes may well have the potential to alter phenotype. Small differences in the methylation level of a locus, such as we have detected by array, could be due to a small methylation change in many cells, or a large methylation change in a small subset of cells. A large methylation change would likely be reflected in a change in gene expression within those particular cells; small changes in methylation might be considered less likely to be associated with a change in gene expression. However, the methylation status of critical CpG dinucleotides at some loci (e.g. within transcription factor binding motifs) can be tightly linked to gene expression [2]; changes at these CpGs could alter gene expression without large methylation changes across the locus. It is also possible that small, widespread changes in methylation induced by a poor intrauterine environment may become magnified over a lifetime and hence accelerate age-associated epigenetic decline [15]; this may go some way to explaining why fetal programming effects are observed later in life.

Fetal programming consistently increases the risk of the metabolic syndrome, despite being induced by a variety of environmental insults. This raises the question of whether specific metabolic genes are targeted by altered nutrition. In our model, methylation changes do not always occur at the same loci in different animals, but affected loci cluster in common gene ontologies. Metabolic ontologies are notable by their absence: rather, the most significant enrichment is seen in gene expression, organ development and cellular development. The fact that control animals (both in our study, and that of Feinberg and Irizzary) also show epigenetic variation within these ontologies suggests that genes in these pathways are “normally” epigenetically plastic; their increased epigenetic variability after supplementation implies that this plasticity (or “metastability”) renders the genes more susceptible to environmental influence. If so, even opposing environmental insults such as gestational undernutrition and overnutrition could produce epigenetic changes in these same pathways. The absence of metabolic ontologies does not necessarily preclude the generation of metabolic phenotypes: changes in organ development, for example, could have indirect metabolic consequences [42].

It has been proposed that adaptation though intrinsic epigenetic diversity may rely ultimately on genetic change within a species [32], but there is no reason to suppose that altered epigenetic states might not become stable in a population (or a subset of a population) without leading to a genetic mutation. The *Lcyc* epimutation of *Linaria vulgaris* represents one example of a potentially adaptive (and reversible) phenotypic change that is purely epigenetic [43]; the epimutation allows the plant to alter its floral symmetry, perhaps in response to environmental cues, and has remained in this species for centuries without effecting a permanent genetic change. Evaluating the heritability of more subtle epigenetic alterations induced by environmental changes, such as those induced by dietary methyl donors in mice, will be key to understanding the impact of early environment on the epigenetic contribution to complex disease risk.

## Methods

### Mice, diets, and tissue

All animals were handled in strict accordance with good practice as defined by the NHMRC (Australia) Statement on Animal Experimentation, and the requirements of NSW State Government legislation. All animal work was approved by the St Vincents/Garvan Animal Ethics Committee (animal research authorities #06/12 and #09/12). C57BL/6 mice were fed *ad libitum* on either (control) NIH-31 diet or (methyl donor supplemented) NIH-31 diet supplemented with (per kg) 15 g of choline, 15 g of betaine, 7.5 g of L-methionine, 150 mg of ZnSO_4_, 15 mg of folic acid and 1.5 mg of vitamin B_12_ (Specialty Feeds, Glen Forrest, Western Australia). Supplementation was commenced two weeks prior to mating founder pairs and continued for six generations; mice to be tested were sacrificed at 5 weeks of age for DNA collection. We extracted DNA from liver tissue, chosen because of its relative cellular homogeneity and high DNA yield.

### Genomic 5-methylcytosine analysis

Genomic 5-methylcytosine (m^5^C) levels in supplemented and unsupplemented mice were assessed using high performance liquid chromatography (HPLC). 1 µg liver genomic DNA was denatured, digested into single nucleotides and dephosphorylated as previously described [44]. HPLC was performed using a method modified from Kovacheva *et al.* [45] with an Atlantis dC18 column (5 µm, 4.6×150 mm) and a 2.5%–16% methanol gradient in 50 mM K_3_PO_4_ (pH 4.5).

### CpG island microarrays and analysis

For CpG island microarray, genomic DNA from supplemented and unsupplemented mice was enriched for the unmethylated fraction as previously described [25]. Briefly, 250 ng liver genomic DNA was subject to HpaII digestion and adaptor ligation followed by a second digestion with McrBC and adaptor-specific PCR. Library preparation was performed in triplicate and replicate libraries pooled for microarray analysis. Libraries were subject to two quality control steps. First, a fraction of each amplified library was analysed by gel electrophoresis and any libraries showing anomalous amplification (low amplicon quantity or unusual size range) were discarded. Second, *in vitro* methylated pCMV DNA and unmethylated pIRES DNA were spiked in to each sample before the McrBC digestion step. After library construction, the control plasmids were PCR amplified and amplicons quantified by densitometry; any libraries showing significant amplification of pCMV (>10% of an unmethylated control sample) or poor amplification of pIRES were discarded.

The DNA libraries were hybridized to Agilent 105K Mouse CpG Island microarrays. Before analysis of microarray data, outliers and low signal intensity features (within 2.6 standard deviations of background) were removed. Data was analysed using Partek Genomics Suite with LOESS normalization and median scaling to zero. We chose to use LOESS normalization because both test and reference samples underwent enrichment, and signals would thus be expected to center around 0, as required by LOESS normalization.

A Shapiro Wilks test in R 2.11.1 [46] was used to confirm that normalized probe signals were normally distributed. Differences in the variance of probe signals between groups were assessed using a Bartlett's test in R 2.11.1, with a *post hoc* analysis comparing the magnitude of probe standard deviation used to identify probes with increased variability.

### Bisulphite methylation analysis

Allelic methylation patterns of selected methylation-variable loci were assessed by bisulphite allelic sequencing [47]. For bisulphite PCR, 2 µg liver genomic DNA was treated with sodium bisulphite using the Epitect Bisulphite kit (Qiagen) and 10% of the reaction was used in each PCR. Amplicons were cloned into pGEM-T and transformed into DH5-α *E. coli* cells, and plasmid DNA from individual colonies was sequenced.

### Motif discovery in methylation-variable regions

For each of the 100 most variable probes in the control samples, we defined the genomic location of the closest known gene's promoter region as 1000 bp upstream and 500 bp downstream of the transcription start site using Galaxy [48] and the mm9 build of the UCSC Genome Browser [49]. As a control we used the 1000 least variable promoters in the control samples. We used GimmeMotifs [50] (version 0.61, using default options and medium motif size, with a randomized genomic background) to discover sequence motifs common to methylation-variable loci. The program Clover (version Jun 12 2006, with default options, and 1000 randomizations and a p-value threshold of 0.05) [51] was used to interrogate whether any of the motifs discovered were enriched in the methylation-variable dataset relative to the 1000 least variable.

### Repeat element associations of methylation-variable regions

Using the same promoter regions as described above, we obtained the GC content of each promoter using the geecee tool from Galaxy, the genomic location of the microsatellites from the microsat track, and the LINE, SINE, LTR, Simple_repeat, Low_complexity, and DNA repeats from the RepeatMasker track, all at UCSC Genome Browser. We compared the distribution of the distance from the TSS to the midpoint of each element for variable *versus* control promoters using a two-sample unpaired t-test, and compared the frequency of these elements using a χ^2^ test, in R 2.11.1 [46].

### Gene ontology of methylation-variable regions

To identify genes associated with methylation-variable probes, the list of array probes with intra-group standard deviation above the 95^th^ percentile of control standard deviations was matched to overlapping annotated genes using Ingenuity Pathways Analysis (IPA) software. Functional analysis of the resulting gene list was performed independently in both IPA and GOStat (http://gostat.wehi.edu.au/), using the array genes and all RefSeq genes (mm9) as reference sets for both analyses.

## Supporting Information

Figure S1 Intrinsic probe variability is not correlated with microarray signal variability. (A) Scatterplots of probe GC content versus microarray probe standard deviation in Control, F1 Methyl and F6 Methyl supplemented mice. Methylation variable probes with standard deviations above the cutoff (dashed line) are colored red (control), blue (F1 methyl) and green (F6 methyl) while those below the cutoff are colored black. The solid red, blue and green lines indicate the average standard deviation values for probes above and below the cutoff in each group. (B) Scatterplots of intrinsic probe variability (standard deviation of reference pool signals across all 15 arrays) versus microarray probe standard deviation in Control, F1 Methyl and F6 Methyl supplemented mice. r values: control, -0.135959; F1 Methyl, -0.155743; F6 Methyl, 0.209424.
(3.18 MB EPS)

Figure S2 Box-and-whisker plots showing sequence features located within the region from −1000 bp to +500 bp relative to the TSS of promoters associated with the 100 most methylation-variable probes (right) versus those associated with the 1000 least methylation-variable probes. (A) GC content; (B) Distribution of repeat elements. Plotted is the distance from the TSS to the midpoint of the element.
(1.50 MB EPS)

Figure S3 Bisulphite allelic sequencing of four methylation variable regions. Microarray signals are shown above, with bisulphite data from the region indicated shown below. Note that an increase in microarray signal indicates *hypo*methylation. The mice with the greatest difference in microarray signal (one control and one methyl donor supplemented) were chosen for bisulphite sequencing. Each square in the bisulphite map represents a CpG; white squares represent unmethylated CpGs while black squares represent methylated CpGs. A row of squares represents the CpGs from an individual sequenced clone. Between 12 and 24 clones were sequenced for each bisulphite map. The overall percentage methylation for each animal is indicated in brackets above the bisulphite map.
(2.60 MB EPS)

Table S1 Methylation variable loci in control, F1 methyl and F6 methyl mice. Listed are all probes with standard deviations above the 95^th^ percentile value in control, F1 methyl, and F6 methyl mice, mapped to nearby genes using Ingenuity Pathways Analysis software.
(1.94 MB XLS)

Table S2 Gene ontologies identified as being significantly enriched in methylation variable genes in control, F1 methyl and F6 methyl mice by GOstat. The highlight color in each table indicates gene ontologies with >10 genes in common.
(0.17 MB XLS)
